# Nanodiamond-Induced Thrombocytopenia in Mice Involve P-Selectin-Dependent Nlrp3 Inflammasome-Mediated Platelet Aggregation, Pyroptosis and Apoptosis

**DOI:** 10.3389/fimmu.2022.806686

**Published:** 2022-04-04

**Authors:** Shih-Che Hung, Lu-Chu Ke, Te-Sheng Lien, Hsuan-Shun Huang, Der-Shan Sun, Chia-Liang Cheng, Hsin-Hou Chang

**Affiliations:** ^1^ Institute of Medical Sciences, Tzu-Chi University, Hualien, Taiwan; ^2^ Department of Molecular Biology and Human Genetics, Tzu-Chi University, Hualien, Taiwan; ^3^ Center for Prevention and Therapy of Gynecological Cancers, Department of Research, Buddhist Tzu Chi General Hospital, Hualien, Taiwan; ^4^ Department of Physics, National Dong Hwa University, Hualien, Taiwan

**Keywords:** nanodiamond induced thrombocytopenia, P-selectin, inflammasome, pyroptosis, platelet regulated cell death, apoptosis, necroptosis, ferroptosis

## Abstract

Nanodiamond (ND) has been developed as a carrier to conduct various *in vivo* diagnostic and therapeutic uses. Safety is one of the major considerations, while the hemocompatibility of ND is not clearly addressed. Here we found that, compared to the other sizes of ND with relatively inert properties, treatments of 50 nm ND induced stronger platelet aggregation, platelet pyroptosis, apoptosis and thrombocytopenia in mice. Blockage treatments of soluble P-selectin, reactive oxygen species (ROS), and Nlrp3 inflammasome inhibitors markedly suppressed such adverse effects, suggesting ND-induced platelet activation and pyroptosis involves surface P-selectin-mediated enhancement of mitochondrial superoxide levels and Nlrp3 inflammasome activation. In addition, challenges of NDs induced less platelet pyroptosis and displayed less thrombocytopenia in P-selectin (*Selp^-/-^*), Nlrp3 (*Nlrp3^-/-^*) and caspase-1 (*Casp1^-/-^*) mutants, as compared to the wild type mice. Blockers of P-selectin, ROS, and Nlrp3 inflammasome pathways could be considered as antidotes for ND induced platelet activation and thrombocytopenia.

## Introduction

With reduced sizes, nanomaterials exert unique physio-chemical properties and are suitable for biomedical applications (1, 2). Among these, nanodiamond (ND) is one of the promising materials attracting researcher’s attentions. With unique spectroscopic properties such as Raman, infrared, and defect-induced color centers fluorescence, ND has been demonstrated as a feasible optical probe in biomedical usages (3–5). In addition, the excellent physical and chemical stability further enable ND as the most biocompatible nanoparticle in the carbon family (5). Early cellular studies have revealed low cytotoxicity of ND. Evidences have shown that ND exerted no toxicity to various cell types, and did not induce cellular reactive oxygen species (ROS) (6, 7). These results and later cell line investigations have concluded that ND is a low cytotoxic material (8).

Despite of these *in vitro* studies, more recent studies have suggested ND is a promising and useful material for drug delivery and bio-labeling (5, 9–11). Because of the potential biomedical applications of ND, and low hemocompatibility limits the use of nanoparticles (12, 13), the hemocompatibility analyses become essential for NDs.

The hemocompatibility of a nanomaterial could be characterized by property on the induction of platelet activation, platelet aggregation, thrombocytopenia and thrombosis after *in vivo* treatments (12, 14–18). Platelets are small anucleate multifunctional blood cells, which involve in many pathophysiological processes including coagulation, thrombosis, inflammation, and innate immunity (18–20). Inflammasomes are caspase-1 containing cytosolic multiprotein complexes, and are activated by pattern recognition receptors in responses to stimulations of pathogen-associated molecular patterns (PAMPs) and danger-associated molecular patterns (21–25). The activated caspase-1 cleaves the immature precursors and leads to the production of mature form of proinflammatory cytokine interleukin-1β (IL-1β), and pore-forming protein gasdermins (26–28). Inflammasomes play critical roles in platelet-mediated inflammation and coagulation (29–31). Expression levels of inflammasome (32) and IL-1β (33–35) in platelets could be up-regulated after stimulations by PAMPs. In addition, platelet inflammasome activation has been revealed in sepsis (36), thrombosis formation (37) and hindlimb ischemia (32) models. Despite the detailed mechanism remains to be further elucidated, activation of inflammasome by dengue virus has been associated with induction of platelet pyroptosis (38). Pyroptosis belongs to the family of regulated cell deaths (RCDs), which include additional cell death pathways such as apoptosis, necroptosis, ferroptosis and autophagy (39–41). Evidences have suggested that RCDs involve in platelet maturation, activation and aggregation (29, 42–45). However, the property of NDs on the induction of platelet cell death remains elusive.

NLR pyrin domain containing 3 (Nlrp3) inflammasome is one of the most studied inflammasomes, sensing a variety of cellular stresses and stimulus, such as ROS, toxins, pathogens, metabolites, nucleic acids, uric acid crystals and nanoparticles (46–50). Several lines of evidences have implicated that over activation of inflammasomes through different pathways in cells can lead to major types of RCDs, including pyroptosis (47), apoptosis (51, 52), necroptosis (52), ferroptosis (53) and autophagy (54, 55). For example, inflammasome activation leads to the maturation and activation of pore-forming protein gasdermins, cell membrane rupture and cell death in pyroptosis (47). Inflammasome activation also leads to apoptosis through Bid and caspase-8 pathways in gasdermin deficient cell models (51). Z-DNA binding protein 1 (ZBP1), a regulator of Nlrp3 inflammasome, was shown to induce pyroptosis, apoptosis, and necroptosis (52). Ferroptosis is associated with inflammasome activation in placental trophoblast cell model of oxidative stress (53). Overexpression of NLRP3 inflammasome components elevated autophagy, and, conversely, silencing of the NLRP3 downregulated autophagy (54). However, these results are obtained from diverse cell models. The regulation networks between inflammasome and these RCDs in a single cell type remains greatly unknown. In addition, the impact of ND treatments on the stimulation of platelet inflammasomes and RCDs remains unclear.

To analyze the hemocompatibility of ND, in this present study, we investigate ND-induced platelet changes *in vitro* and thrombocytopenia *in vivo*. The analyses data revealed that ND induces platelet aggregation is associated with P-selectin-dependent enhanced ROS-medicated activation of Nlrp3 inflammasome and subsequently platelet pyroptosis. Challenges of NDs induced less platelet cell death in P-selectin (*Selp^-/-^*), Nlrp3 (*Nlrp3^-/-^*) and caspase-1 (*Casp1^-/-^*) null mice as compared to the wild type mice. Treatments of inhibitors against P-selectin, ROS and Nlrp3 inflammasome pathways ameliorated both ND-induced platelet activation *in vitro* and ND-induced thrombocytopenia in mice. These results collective suggested that ND-induced Nlrp3 inflammation activation is one of the initiation steps leading to the platelet activation and thrombocytopenia *in vivo*. Administrations of ND with lower doses are helpful to reduce such platelet-related adverse effect. Related regulatory pathways in ND-stimulated platelets are discussed.

## Materials and Methods

### Chemicals and Nanomaterials

The chemicals used in this study were purchased from Sigma-Aldrich (St. Louis, MO, USA). To prepare the stock solutions of 10 mg/mL TiO_2_ (5 and 60 nm; Nanostructured & Amorphous Materials, Katy, TX, USA), 10 mg/mL NDs (5-200 nm; Kay Diamond Products, Boca Raton, FL. USA) (56, 57), and red fluorescent NDs (50 nm; brFND-50, nitrogen-vacancy NV centers per particle > 100, FND Biotech, Taipei, Taiwan) (58, 59), the nanoparticles (NPs) were dispersed in distilled deionized water under sonication (80 W/L, 46 kHz) for 20 min. Test NP solutions were prepared immediately before use by dilution of the stock solutions with distilled deionized water and sonication (80 W/L, 46 kHz) for 20 min (60).

### Experimental Mice

Wild type male C57BL/6J mice (8–12 wk old) were obtained from the National Laboratory Animal Center (Taipei, Taiwan). Gene knockout mice with a C57BL/6J background, including *Nlrp3^-/-^* and *Casp1^-/-^* (61), were kindly provided by the Centre National de Recherche Scientifique (Orléans, France) (61–63). C57BL/6J male mice (8–12 wk old) deficient in P-selectin (B6; 129S2-*Selp^tm1Hyn^*/J) (*Selp^-/-^*) (19, 64, 65) were purchased from the Jackson Laboratory (Maine, USA). All animals were maintained in a specific-pathogen-free (SPF) facility in the Laboratory Animal Center of Tzu Chi University (Hualien, Taiwan).

### Ethics Statement

Animal experiments in this study were conducted in agreement with the National (Taiwan Animal Protection Act, 2008) directive for the protection of laboratory animals. All experimental protocols for examining experimental animals were approved by the Animal Care and Use Committee of Tzu-Chi University, Hualien, Taiwan (approval ID: 108067).

### Blood and Platelet Isolation and Parameter Analyses

Collected mouse blood samples were transferred into polypropylene tubes containing anticoagulant acid-citrate-dextrose solution (38 mM citric acid, 75 mM sodium citrate, and 100 mM dextrose) (64, 65). Washed platelets were prepared as previously described (19, 38). Platelet counts of mice were measured using a hematology analyzer (KX-21N; Sysmex, Kobe, Japan) (64–66).

### *In Vivo* Analyses: The Induction and Rescue of Thrombocytopenia in Mice

Various sizes (5, 50, 100, 200 nm) of NDs, or different doses (0.3125, 0.625, 1.25 mg/kg) of 50 nm NDs were injected into mice intravenously. Platelet counts were analyzed 1, 4, 24 and 72 h later after ND treatments using a hematology analyzer (KX-21N; Sysmex). To perform rescue, regents were pretreated before administration of NDs (NAC 300 mg/kg, Sigma-Aldrich; MitoTEMPO 0.1 mg/kg, Sigma-Aldrich; OLT1177 50 mg/kg, Cayman Chemical, Ann Arbor, MI, USA; soluble recombinant P-selectin, rP-sel, 0.24 mg/kg, R&D Systems, Minneapolis, MN, USA; Z-WEHD-FMK, 7.5-750 μg/kg, R&D Systems; Z-DEVD-FMK, 6.5-65 μg/kg, R&D Systems), and then the platelet counts were then analyzed additional 1 h after ND treatments.

### *In Vitro* Analyses: Platelet Regulated Cell Death and Mitochondrial Superoxide

Proteins (1 mg/mL), including bovine serum albumin (BSA) (Sigma-Aldrich), C-type lectin domain family 2 [CLEC2, a gift from Professor Shie-Liang Hsieh, Genomics Research Center, Academia Sinica, Taipei, Taiwan (67)], toll-like receptor 4 (TLR4; R&D Systems), rP-sel (R&D Systems), coated fluorescent silica beads (1 mg/mL, Bangs Laboratories, Fishers, IN, USA; Alex488-goat-anti-mouse antibody pre-coated before aforementioned protein coating) and red fluorescent NDs (1 mg/mL, brFND-50, FND-Biotech) were used to analyze ND protein binding. After incubation of NDs with different proteins for 1 h, the ND-protein complexes were analyzed using flow cytometer. To determine ND-induced platelet aggregation, mouse platelets (5 × 10^7/^mL) were treated with NDs (30 μg/mL). After 1 h, the aggregated populations were analyzed using flow cytometry (gating in **Figure S1**). To analyze ND induced platelet cell death, washed mouse platelets from wild type and mutant (*Selp^-/-^*, *Nlrp3^-/-^* and *Casp1^-/-^*) mice were incubated with ND for 1 h in a shaker (20 rpm, 25°C) and then subjected to analyses by flow cytometers [Gallios, Beckman Coulter, Brea, CA, USA, and FACScalibur, BD Biosciences, San Jose, CA, USA (64, 65)] analyses after washed with PBS. Various regulated cell death (RCD) responses, including apoptosis (CaspGLOWTM Red Active Caspase-3 Staining Kit, BioVision, Milpitas, CA, USA), autophagy (Cyto-ID™ Autophagy Detection Kit, Enzo Life Sciences, Farmingdale, NY, USA), ferroptosis (C11 BODIPY 581/591, Cayman Chemical, Ann Arbor, MI, USA), necroptosis (RIP3/B-2 alexa Fluor 488, Santa Cruz Biotechnology, Santa Cruz, CA, USA), pyroptosis (Caspase-1 Assay, Green, ImmunoChemistry Technologies, MI, USA), and live/dead cell labeling (Zombie NIR Fixable Viability Kit, Biolegend, San Diego, CA, USA), were analyzed using respective cell labeling reagents (30 min in PBS). Notably, to avoid detecting those RCD signals not contributing by the ND treatments (e.g. those RCDs elicited by purification and manipulation processes), Zombie-live/dead cell labeling (30 min) should be performed immediately after ND treatments, and before the subsequent RCD signal staining (30 min); and then the RCD pattern only analyzing on dead-cell population indicating by Zombie-live/dead staining. Blockers and inhibitors were used to address the involvements of specific pathways in platelets from wild type mice (Z-WEHD-FMK, 10 μM, R&D Systems; Z-DEVD-FMK, 10 μM, R&D Systems; OLT1177, 10 μM, Cayman Chemical; NAC 150 ng/mL, Sigma-Aldrich; MitoTEMPO, 1 μM, Sigma-Aldrich; P-selectin: rP-sel, 100 ng/mL R&D Systems; 30 min pretreatments before addition of ND). To analyze the induction of mitochondrial superoxide, MitoSOX™ Red mitochondrial superoxide indicator was used (Thermo Fisher Scientific; 30 min in PBS). Carboxyfluorescein succinimidyl ester (CFSE, Sigma-Aldrich) and CellTracker Blue Dye (ThermoFisher Scientific, Waltham, MA, USA) were used to label mouse platelets for flow cytometry and microscopy analyses.

### *In Vitro* Analyses: Confocal Microscopy Analysis on the Morphology of Platelet Aggregates

A confocal microscope (C2+, Nikon, Tokyo, Japan) was employed on the analysis of platelet aggregate morphology. Same conditions of treatment dosage for ND and cell death inhibitors were applied in the confocal microscopy as the conditions used in the platelet cell death analyses. To distinguish populations of platelets, NDs and platelet-ND aggregates, CellTracker Blue Dye (ThermoFisher Scientific) labeled mouse platelets, and red fluorescent 50 nm NDs (brFND-50, FND Biotech) were used in this experiment. The counts of platelet aggregates per field (> 400 pixels) and the total platelet aggregate area (pixels) per field were analyzed using ImageJ software (version 1.32; National Institutes of Health, USA) (38, 68).

### Neutrophil Extracellular Traps Formation (NETosis)-Related Analyses

According to previously reported methods (69), neutrophils were purified from mouse blood samples using Ficoll-Paque (Ficoll-Paque Plus, 1.077 g/mL, GE Healthcare, Chicago, IL, USA) and dextran (Sigma-Aldrich) sedimentation (3% w/v) density gradient centrifugation and red blood cell lysis. A flow cytometer (Gallios, Beckman Coulter, Brea, CA, USA) and a fluorescent anti-citrullinated histone H3 (CitH3) antibody (Abcam, Cambridge, UK) were used to investigate the neutrophil expression of NETosis marker CitH3 after treatments of supernatants from platelets or platelets plus NDs. To prepare the platelet supernatants, inhibitors (Z-WEHD-FMK, 10 μM, R&D Systems; Z-DEVD-FMK, 10 μM, R&D Systems; OLT1177, 10 μM, Cayman Chemical; NAC 150 ng/mL, Sigma-Aldrich; MitoTEMPO, 1 μM, Sigma-Aldrich; P-selectin: rP-sel, 100 ng/mL R&D Systems; 30 min pretreatments before addition of ND) were used to block ND-induced platelet activation and cell death. After treatments with or without NDs and inhibitors, platelet supernatants were harvested by centrifugation (2.5 x 10^4^ g, 10 min; Benchtop Centrifuge, ThermoFisher Scientific) to remove platelets and NDs. Peptidyl arginine deiminase 4 (PAD4) inhibitor GSK484 (10μM, Sigma–Aldrich, St. Louis, MO, USA) was used to block neutrophil NETosis *in vitro* and *in vivo* as described (69).

### Statistical Analyses

The means, standard deviation (SD), and statistics of the quantifiable data were calculated using Microsoft Office Excel 2003, SigmaPlot 10, and SPSS 17, respectively. Unless specified, the significance of the data was examined using one-way ANOVA, followed by the *post hoc* Bonferroni-corrected *t* test. A probability of type 1 error (α = 0.05) was recognized as the threshold for statistical significance.

## Results

### Different Sizes of NDs Induced Different Levels of Platelet-Count Suppression in Mice

**Figure 1:**
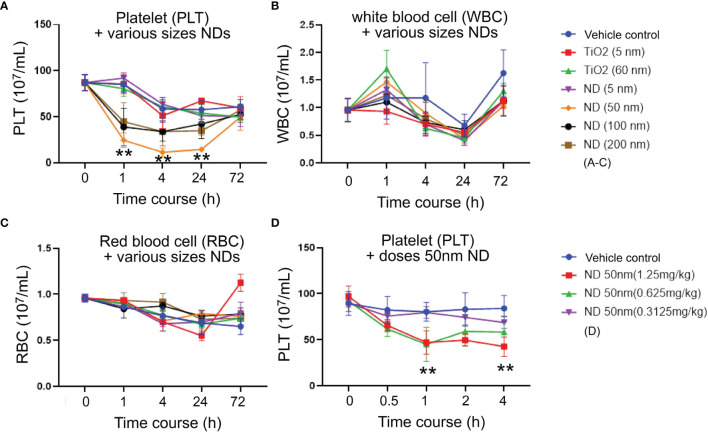
ND-induced thrombocytopenia in mice. **(A)** Platelet (PLT) counts, **(B)** white blood cell (WBC) counts, **(C)** red blood cell (RBC) counts, of wild type C57Bl/6J mice challenged by TiO_2_ (5 and 60 nm; 1.25 mg/kg) and various sizes of ND (5-200 nm; 1.25 mg/kg) nanoparticles. **(D)** Platelet counts of wild type C57Bl/6J mice challenged by various doses (0.625-1.25 mg/kg) of 50 nm ND nanoparticles. n = 6 (three experiments with two mice per group). **P < 0.01, vs. 0 h groups.

To investigate how ND sizes influence the blood cell counts, various sizes (5 nm, 50 nm, 100 nm and 200 nm; **Figures 1A–C**) of NDs were intravenously injected into mice. Here we found that, compared to red blood cell and white blood cell counts, platelet counts displayed more divergence outcomes when treated with different sizes of NDs (**Figures 1A** vs. **B, C**), in which the 50 nm ND induced more severe thrombocytopenia as compared to nanoscaled titanium dioxide (TiO_2_; 5 nm and 60 nm), and the other sizes of ND (**Figure 1A**, 50 nm ND vs. 5 nm, 100 nm, and 200 nm groups). Different doses (0.3, 0.6, 1.25 mg/kg) of 50 nm NDs were further injected into the mice to evaluate the dosage effect. Analysis data revealed that only treatments with low dose (0.3 mg/kg) did not displayed obvious effects, while treatments with doses higher than 0.6 mg/kg (0.625 and 1.25 mg/kg) of 50 nm ND caused markedly lower platelet counts in mice (**Figure 1D**).

### Pyroptosis and Apoptosis Are Two Major Cell Death Pathways of Platelets Treated With 50 nm NDs

**Figure 2:**
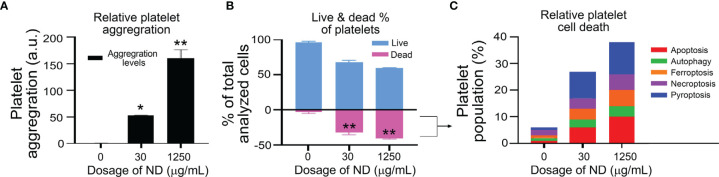
ND-induced platelet cell death and aggregation *in vitro*. **(A, B)** Treatments of 50 nm NDs induced dose-dependent (0, 30, 1250 μg/mL) platelet aggregation **(A)** and cell death **(B)**, as measured by flow cytometry (**A**, gating in **Figure S1**), and Zombie NIR live/dead analysis kit **(B)**, respectively. **(C)** We observed the ND treatments induced multiple regulated cell death pathways (RCDs) in the dead cell population of mouse platelets. n = 6 (3 experiments with 2 samples per group). *P < 0.05, **P < 0.01, vs. vehicle control (0 μg/mL) groups.

Evidences have suggested that platelet activation and aggregation involve RCD processes of platelets (42–45). However, whether platelet RCDs also involve in ND-induced platelet aggregation and thrombocytopenia is not clearly addressed. In addition, according to our previous findings, one cell-death inducer may trigger multiple RCDs in a specific cell type, such as platelet (38). The compositions of multiple RCDs are identified and described as cell-type-specific RCD patterns (CTS-RCDPs) (38, 62, 69). Accordingly, we would like to investigate 50 nm ND-induced RCD and CTS-RCDP in platelets. Those most described RCD pathways (40), which include pyroptosis, necroptosis, ferroptosis, apoptosis, and autophagy, were analyzed using flow cytometry approach following previously described methods (38, 62, 69). We found that 50 nm NDs induced platelet cell death levels are associated with platelet aggregation levels in a dose-dependent manner (**Figure 2A**, aggregation levels, gating in **Figure S1**; **Figure 2B**, dead cell populations). Flow cytometry analyses of CTS-RCDP revealed that treatments with 50 nm NDs induced considerable higher levels of pyroptosis and apoptosis as compared to the other analyzed RCDs in platelet death cell population (**Figure 2B**, dead cell population; **Figure 2C**, 30 and 1250 μg/mL ND groups, indicated ND-induced platelet CTS-RCDP; gating and calculation of CTS-RCDP in **Figure S2**).

### Inhibitors Against P-Selectin, Nlrp3 Inflammasome Pathways Suppressed ND-Induced Platelet Aggregation, and Cell Death *In Vitro*

**Figure 3:**
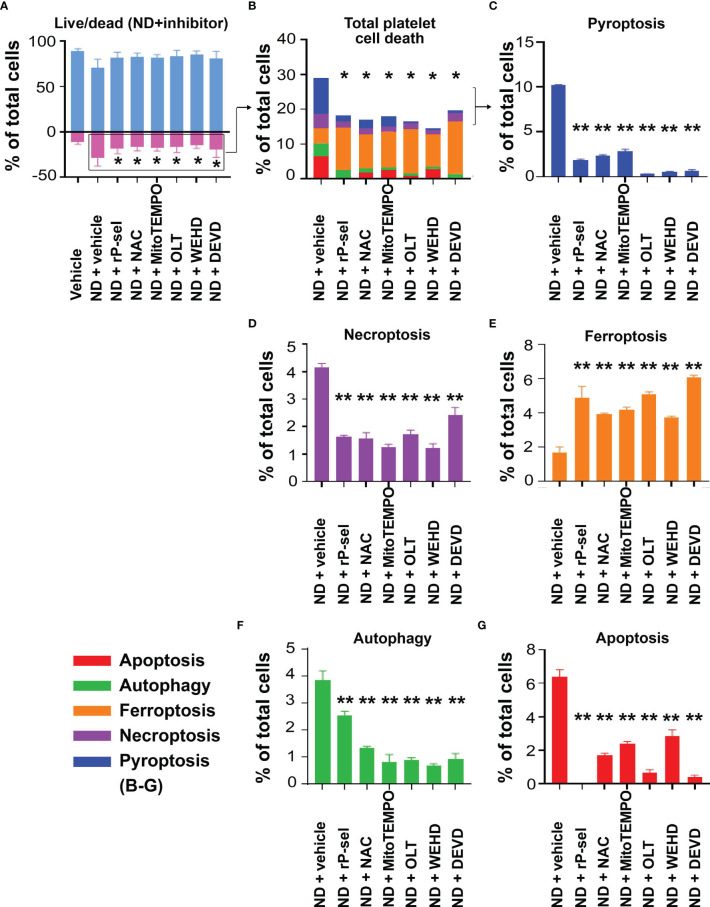
P-selectin, Nlrp3, caspase-1 and caspase-3 inhibitors protect platelets from ND-induced pyroptosis and apoptosis. Treatments with competitive P-selectin inhibitor rP-sel (100 ng/mL), ROS inhibitor NAC (150 μg/mL), mitochondria-targeted antioxidant MitoTEMPO (10 μMu;), Nlrp3 inhibitor OLT1177 (OLT, 10 μM), caspase 1 inhibitor Z-WEHD-FMK (WEHD, 10 μM) and caspase 3 inhibitor Z-DEVD-FMK (DEVD, 10 μM) rescued ND-induced platelet cell death **(A, B)**. By dividing total cell death **(B)** into respective RCDs **(C–G)**, we found that pyroptosis and apoptosis are the top 2 RCDs induced by ND challenges. Additional treatments with rP-sel, NAC, MitoTEMPO, OLT1177, Z-WEHD-FMK and Z-DEVD-FMK, all markedly rescued ND-induced platelet pyroptosis **(C)**, apoptosis **(G)**, necroptosis **(D)** and autophagy **(F)** levels. Despite overall platelet survival rate increased after the inhibitor treatments, the ferroptosis levels exacerbated **(E)**. n = 6 (3 experiments with 2 samples per group), *P < 0.05, **P < 0.01, vs. vehicle groups.

**Figure 4:**
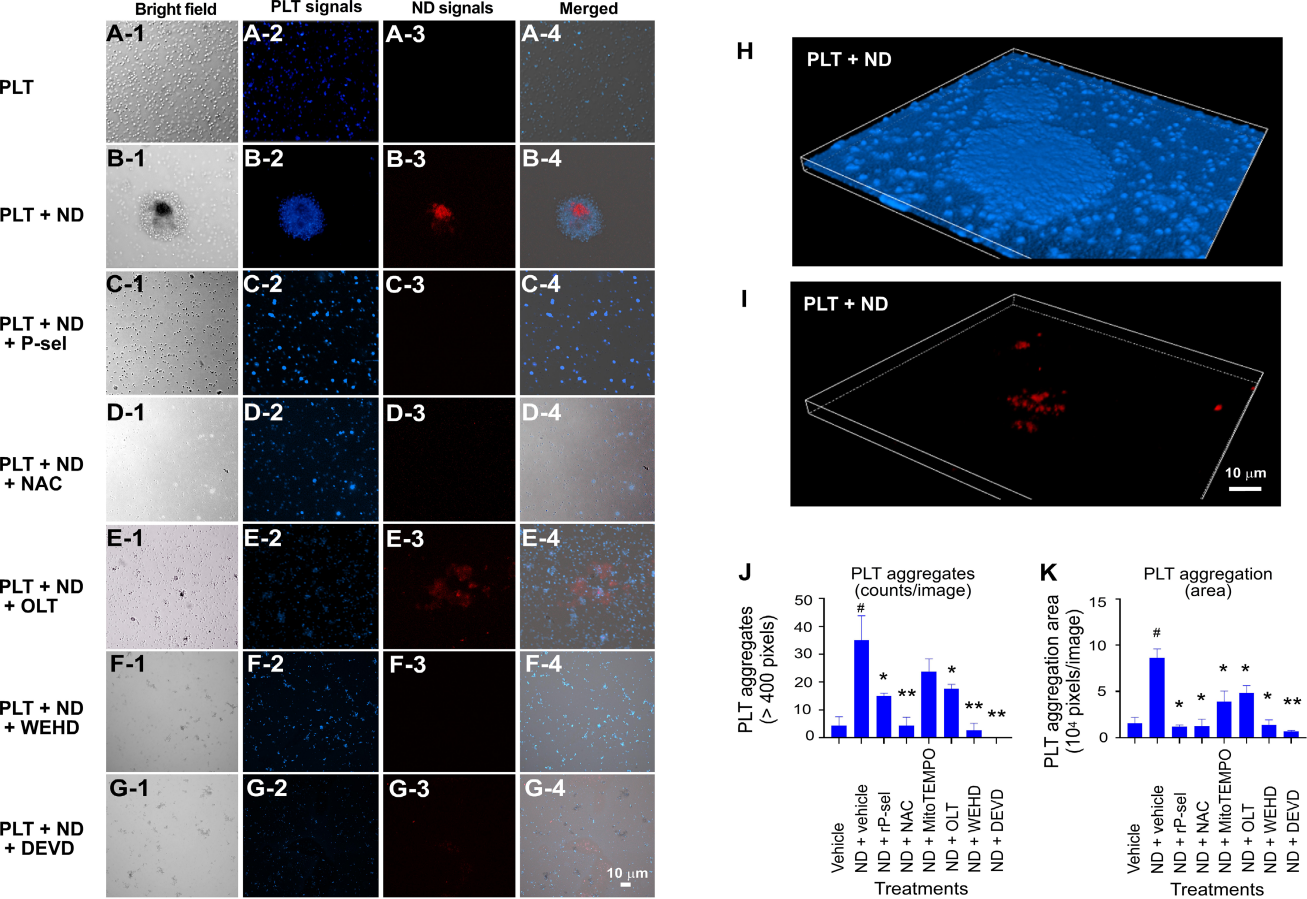
Confocal microscopy analyses on the morphology of ND-induced platelet aggregates. **(A–I)** Example images of platelet (PLT) aggregates that were induced by treatments of ND, with or without additional treatments of inhibitors (rP-sel, NAC, OLT1177, Z-WEHD-FMK and Z-DEVD-FMK) were shown. CellTracker Blue Dye labeled mouse platelets, and red fluorescent 50 nm NDs (NV center > 100 per particle, FND Biotech) were employed in this experiment. **(J, K)** Quantitative analyses revealed that ND treatments markedly enhanced the platelet aggregate counts (those > 400 pixels) **(J)**, and area **(K)**. All tested inhibitors (rP-sel, NAC, MitoTEMPO, OLT1177, Z-WEHD-FMK and Z-DEVD-FMK) suppressed ND-induced aggregation **(K**, area; and **J**, counts; except MitoTEMPO**)**. n = 6 (2 experiments with 3 mice per group). ^#^ *P* < 0.05 vs. vehicle groups. ^*^ *P* < 0.05, ^**^ *P* < 0.01, vs. ND + vehicle groups. Example images of 3D morphology of ND-induced platelet aggregate are highlighted **(H, I**; similar result referred to **Supplementary Video S1**). Scale bars: **(A–G)**, 10 μm (G-4); **(H, I)**, 10 μm **(I)**.

To investigate potential therapeutic interventions through suppression of pyroptosis and apoptosis, ROS inhibitors (N-acetyl-l-cysteine [NAC], mitochondria-targeted antioxidant MitoTEMPO (62, 70)), Nlrp3 inhibitor OLT1177, inflammasome/caspase1 inhibitor Z-WEHD-FMK (38, 62, 69), caspase-3 inhibitor Z-DEVD-FMK (71), were used in the following experiments. Our parallel experiments revealed that P-selectin, an adhesion receptor expressing on the surfaces of activated platelets and endothelial cells, displayed markedly higher ND-binding property as compared to various control proteins, including known pattern recognition receptors of platelets, such as toll-like receptor 4 (TLR4) and C-type lectin domain family 2 (CLEC2) (**Figure S3**). Consistently, platelets from wild type (*Selp^+/+^*) mice displayed relatively higher ND-binding property as compared to the P-selectin-deficient platelets from the *Selp^-/-^* mutant mice (**Figure S4**). In addition, when compared to BSA, soluble recombinant P-selectin (rP-sel) treatments drastically suppressed 50 nm NDs induced platelet cell death as compared to the BSA-treated controls (**Figure S5**). Accordingly, here we used rP-sel as additional platelet cell death inhibitor in the following experiments. Analyses results revealed that treatments of rP-sel, NAC, MitoTEMPO, OLT1177, Z-WEHD-FMK and Z-DEVD-FMK markedly reduced ND-induced platelet total cell death (**Figure 3A**). By dividing total death cell population (**Figure 3A**, dead-cell population) into respective RCD cell populations (**Figures 3B–G**; **Figure S6**, specific RCD inducers induced platelet cell death, positive controls of RCD analyses), we found that inhibitors rP-sel, NAC, MitoTEMPO, OLT1177, Z-WEHD-FMK and Z-DEVD-FMK suppressed RCDs, including pyroptosis, apoptosis, necroptosis, autophagy, except ferroptosis (**Figures 3C, D, F, G**). To investigate whether the platelet aggregation is associated with the induction and reversal of platelet cell death, the morphology of ND-induced platelet aggregation was further analyzed using confocal microscopy under conditions with or without the inhibitor treatments. In agreement with the cell death analyses, NDs are able to induce platelet aggregation, and such platelet aggregates are markedly suppressed by treatments of cell death inhibitors, which include rP-sel, NAC, MitoTEMPO, OLT1177, Z-WEHD-FMK and Z-DEVD-FMK (**Figures 4A–I**, example images; **Figures 4J, K**, quantitative results; video S1, an example 3D structure of ND-platelet aggregates). These results suggested that ND-induced platelet aggregation is associated with ND-induced cell death.

### ND Induced Less Pyroptosis and Apoptosis in Platelets From *Selp^-/-^*, *Nlrp3^-/-^* and *Casp1^-/-^* Mutants, as Compared to the Same Treatments to Platelets From Wild Type Mice

**Figure 5:**
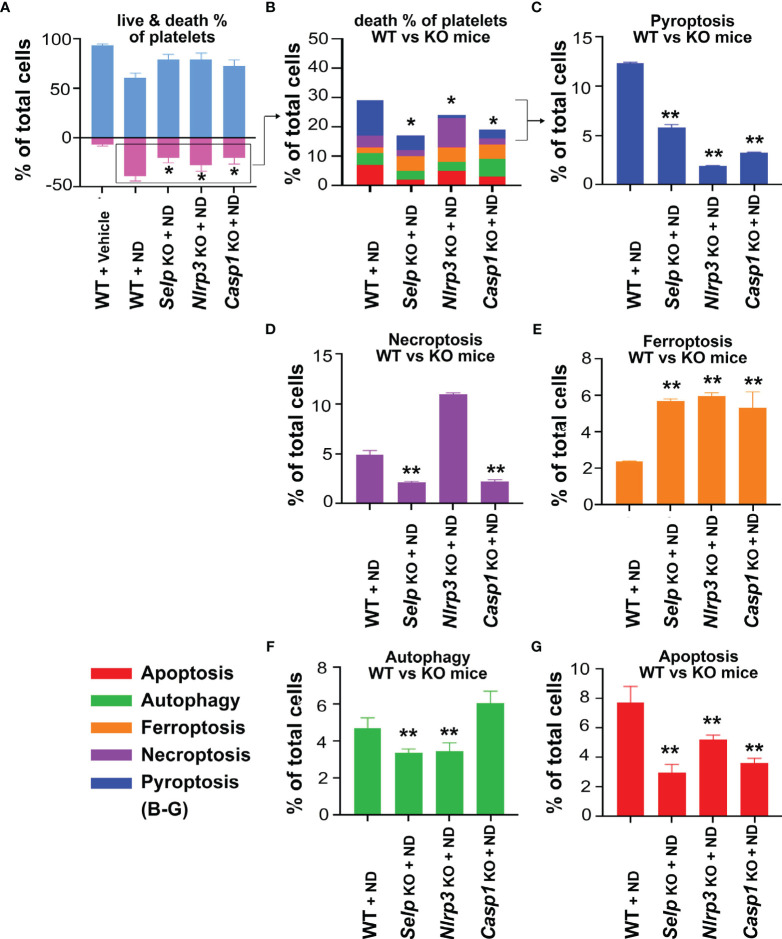
P-selectin, Nlrp3 and caspase-1 deficiencies protect platelets from ND-induced pyroptosis and apoptosis. **(A, B)** Compared with wild type (WT) controls, P-selectin (*Selp^-/-^*), Nlrp3 (*Nlrp3^-/-^*) and caspase 1 (*Casp1^-/-^*) deficient platelets displayed less cell death levels in response to ND treatments. **(C–G)** Consistent with the cell death analysis, platelets from P-selectin (*Selp^-/-^*), Nlrp3 (*Nlrp3^-/-^*) and caspase 1 (*Casp1^-/-^*) deficient mice displayed less pyroptosis and apoptosis, the 2 major RCDs, levels in response to ND challenges. n = 6 (2 experiments with 3 mice per group), *P < 0.05, **P < 0.01, vs. WT groups.

To further verify whether platelet P-selectin and Nlrp3 inflammasome (Nlrp3 and caspase 1) pathways indeed involve in ND induced platelet cell death, live/dead and CTS-RCDP status were analyzed using platelets from *Selp^-/-^*, *Nlrp3^-/-^* and *Casp1^-/-^* mutants. In agreement with the inhibitor experiments, 50 nm ND induced less pyroptosis and apoptosis levels in platelets from *Selp^-/-^*, *Nlrp3^-/-^* and *Casp1^-/-^* mutants, as compared to the same treatments to platelets from wild type mice (**Figure 5**). These results suggested that P-selectin and Nlrp3 inflammasome pathways indeed involve in ND-induced pyroptosis and apoptosis.

### Inhibitors Against P-Selectin, ROS and Nlrp3 Inflammasome Pathways Suppressed Platelet Aggregation and Mitochondria Superoxide Burden *In Vitro*

**Figure 6:**
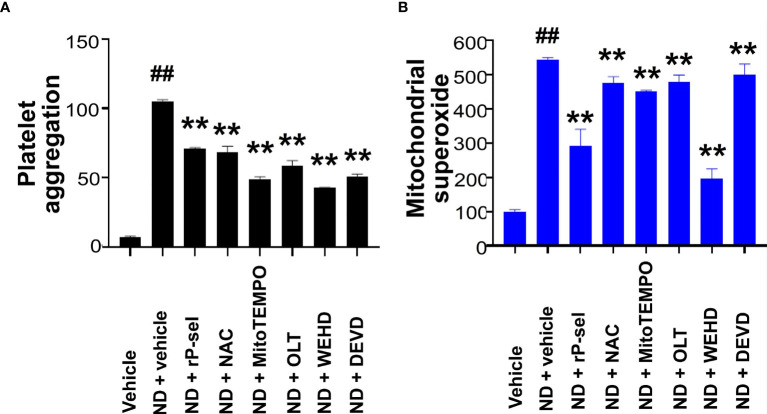
P-selectin, Nlrp3, caspase-1 and ROS inhibitors protect platelets from ND-induced activation. ND-induced platelet activation, including **(A)** platelet aggregation, and **(B)** mitochondrial superoxide levels, could be suppressed with the treatments with P-selectin, ROS inhibitors, Nlrp3, caspase-1 and caspse-3 inhibitors [rP-sel (100 ng/mL), OLT1177 (OLT, 10 μM), Z-WEHD-FMK (WEHD, 10 μM), Z-DEVD-FMK (DEVD, 10 μM), NAC (150 μg/mL), and MitoTEMPO (1 μM)], respectively **(A, B)**. **(A)** ND + vehicle groups were normalized to 100%; **(B)** vehicle groups were normalized to 100%. n = 6 (2 experiments with 3 mice per group), ^##^ *P* < 0.05, vs. respective vehicle groups; **P < 0.01, vs. respective ND + vehicle groups.

Because Nlrp3 inflammasome-mediated pyroptosis is a major RCD involved in 50 nm ND-induced platelet defects, we further investigated whether the suppression of platelet Nlrp3 inflammasome through inhibitor treatments is sufficient to ameliorate 50 nm ND-induced abnormal platelet activation. Here we found that 50 nm ND-induced platelet aggregation, and increased mitochondrial superoxide levels (**Figure 6**). Superoxide is a powerful cell-damaging ROS, which is produced in mitochondria by electrons leaking from the electron transfer system (72, 73). Upregulated mitochondrial superoxide indicated increased levels of cellular oxidative stress and mitochondrial burden (72, 73). Consistent with the platelet cell death analyses (**Figure 3**), ND treatments with additional P-selectin (rP-sel), Nlrp3 inflammasome (OLT1177 and Z-WEHD-FMK), apoptosis (Z-DEVD-FMK), and ROS [NAC; and MitoTEMPO, a mitochondria targeted antioxidant (62, 70)] inhibitors treatments, ameliorated such ND-induced platelet aggregation and mitochondria superoxide levels (**Figure 6**).

### Inhibitors Against P-Selectin, ROS, Nlrp3 Inflammasome Pathways Suppressed Platelet Aggregation, Pyroptosis and Apoptosis *In Vivo*

**Figure 7:**
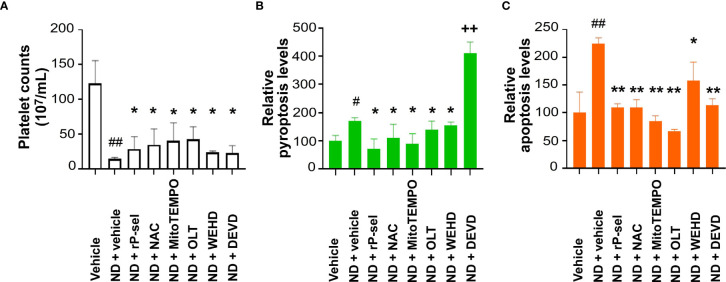
Treatments of P-selectin, ROS, Nlrp3, caspase-1, and caspase-3 inhibitors ameliorate ND-induced thrombocytopenia in mice. Treatments with P-selectin, ROS, Nlrp3, caspase-1, and caspase-3 inhibitors [rP-sel (0.24 mg/mL), OLT1177 (OLT, 50 mg/kg), NAC (300 mg/kg), and MitoTEMPO (0.1 mg/kg), Z-WEHD-FMK (ZWEHD, 750 μg/kg), Z-DEVD-FMK (DEVD, 6.5 μg/kg)], ameliorated ND (50 nm; 1.25 mg/kg)-induced thrombocytopenia **(A)**, and platelet pyroptosis **(B)** and apoptosis **(C)** levels in C57BL/6J mice. **(B, C)** Vehicle groups were normalized to 100%. n = 6, (2 experiments with 3 mice per group). ^#^ *P* < 0.05, ^##^ *P* < 0.01, vs. respective vehicle groups; *P < 0.05, **P < 0.01 significantly lower, vs. respective ND + vehicle groups; ^++^ *P* < 0.01 significantly higher, vs. ND + vehicle groups.

*In vivo* mouse experiments further revealed that, in agreement with the *in vitro* analyses, treatments with P-selectin (rP-sel), Nlrp3 inflammasome (OLT1177 and Z-WEHD-FMK), apoptosis (Z-DEVD-FMK), and ROS (NAC and MitoTEMPO) inhibitors markedly ameliorated ND-induced thrombocytopenia (**Figure 7A**), and platelet pyroptosis (**Figure 7B**, except Z-DEVD-FMK) and apoptosis (**Figure 7C**) levels in C57BL/6J mice. These results suggested that 50 nm ND induces platelet aggregation and platelet cell death, involve P-selectin, and Nlrp3 inflammasome mediated enhancement of mitochondrial oxidative stress, pyroptosis and apoptosis.

### Inhibitors Against P-Selectin, ROS, Nlrp3 Inflammasome and Caspase-3 Pathways Suppressed ND-Induced Platelet-Enhanced Neutrophil Extracellular Trap Formation

**Figure 8:**
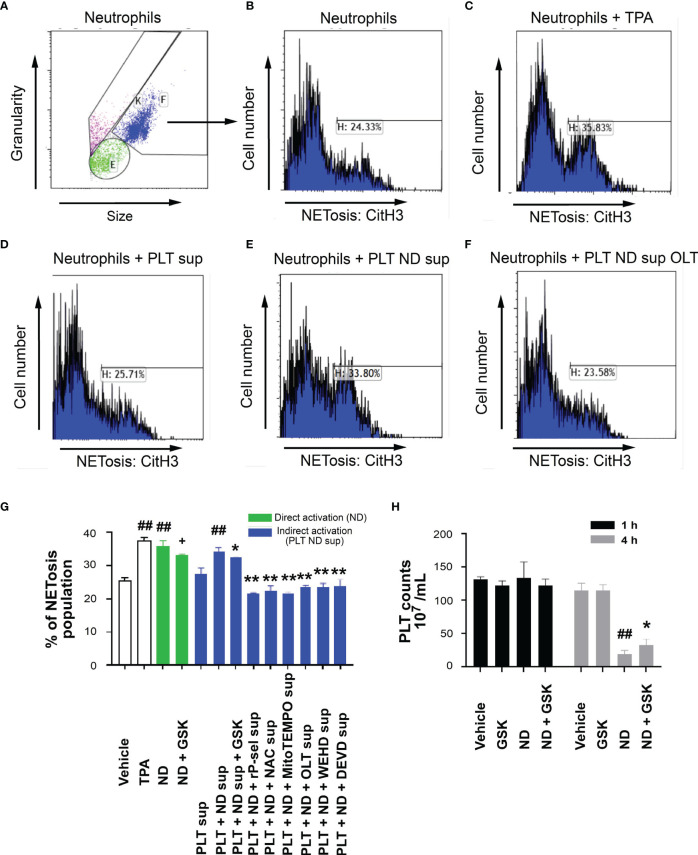
Reversal of ND-induced thrombocytopenia through suppression of NETosis in mice. Flow cytometry gating of NETosis (citrullinated histone H3, CitH3 staining) levels of mouse neutrophils treated with vehicle **(A, B)**, 12-O-tetradecanoylphorbol-13-acetate **(C**, TPA, a positive control NETosis inducer; 2 nM**)**, and supernatants from 50 nm NDs (30 μg/mL) activated (30 min) wild mice platelets (2 × 10^6^) (PLT ND sup) **(E)** with or without additional inhibitor **(F**, OLT**)** pretreatments (30 min), as compared to the NETosis induced by none-activated platelet supernatants **(D**, PLT sup**)**. **(G)** The quantified results indicated that ND can induce NET formation directly (green columns). ND treatments can also enhance NETosis indirectly through soluble factors released from ND-activated platelets **(G**, PLT+ND sup**)**, and such this “PLT+ND sup”-induced NETosis could be suppressed by treatments of additional inhibitors such as GSK484, rP-sel, NAC, MitoTEMPO, OLT1177, Z-WEHD-FMK and Z-DEVE-FMK **(G**, blue columns**)**. **(H)** Treatments NETosis inhibitor GSK484 ameliorated ND-induced thrombocytopenia. **(G, H)** ^##^ *P* < 0.01 vs. vehicle groups. **(G)** ^+^ *P* < 0.05, vs. ND groups; *P < 0.05, **P < 0.01, vs. “PLT + ND sup” groups. **(H)** *P < 0.05, vs. ND groups. n = 6 (2 experiments with 3 mice per group).

Flow cytometry analyses were employed to further investigate whether ND-induced platelet cell death involves in ND-induced NETosis. In agreement with previous reports (74–76), we found that ND-treatments directly induced mouse neutrophil NETosis (**Figures 8A–F** gatings; **Figure 8G** quantitative results). Intriguingly, after stimulation and removal of NDs, the supernatants form ND-activated platelets (PLT+ND sup) also induced comparable levels of NETosis (**Figure 8G**, ND groups vs. PLT+ND sup groups). In addition, the capability of the PLT+ND sup to induce NETosis could be suppressed by treatments of platelet cell death inhibitors, including rP-sel, NAC, MitoTEMPO, OLT1177, Z-WEHD-FMK and Z-DEVD-FMK, during ND stimulation (**Figure 8G**). This suggests that, in addition to direct stimulation of neutrophils, ND can also induce NETosis indirectly through induction of platelet activations. Platelet counts of ND-challenges of mice, with or without NETosis inhibitor GSK484 treatments, were further evaluated to investigate whether NETosis might involve in ND-induced thrombocytopenia. Data revealed that GSK484 treatments markedly rescued ND-induced low platelet counts (**Figure 8H**). These results suggested that there is a feedforward regulation between platelets and neutrophils: ND-activated platelets release soluble factors induced NETosis, and NETosis further exacerbate ND-induced thrombocytopenia.

## Discussion

Hemocompatibility of blood-contacting nanomaterials is one of the most important criteria for their successful *in vivo* applicability (12, 17, 18). Among coagulation cascades, platelet activation and aggregation play central roles in determining the hemocompatibility of nanomaterials (18). As more *in vivo* applications of ND have been reported (5, 9–11), in this present study, we analyzed the impact of ND treatments on the stimulation of platelet aggregation and cell death. Analyses results revealed that among the various sizes (5-200 nm) of detonation NDs, with unknown reasons, treatments of 50 nm NDs induced highest level of platelet aggregation and thrombocytopenia, *in vitro* and *in vivo*. Complex factors may affect the hemocompatibility of nanomaterials *in vivo*, such as nanomaterial interaction and activation with respective types of blood cells and coagulation cascades (12, 13, 17). Thus, the mechanism of how 50 nm NDs displayed highest hemocompatibility-associated adverse effects is worthy of further investigated. In addition, here we found that NDs induce platelet cell death dependent aggregation, because pyroptosis and apoptosis inhibitors block the platelet aggregation *in vitro* and thrombocytopenic responses *in vivo*. These results suggested that suppression of platelet pyroptosis and apoptosis could be a useful method to manage the hemocompatibility-associated adverse effects of ND.

Our previous findings have shown that a single domain of dengue virus protein (envelope protein domain III; DENV-EIII) can elicit various types of RCDs in different cell types including platelets (38, 62, 69). With more complexed compositions, ND surfaces were reported to have evenly distributed high levels of phenols, pyrones, and sulfonic acid groups, as compared to hydroxyl and epoxide groups that are present only on some areas of the surfaces (77). Therefore, it is reasonable that ND can induce various RCDs in platelets. It is yet unclear why single reagent, such as ND and dengue virus envelope protein (38), can induce different types of RCDs in platelets. Cell death pathways have long been considered to regulate in independently; while it is currently clear that pyroptosis, necroptosis, and apoptosis are tightly connected and can cross-regulate each other (78). For example, in the absence of gasdermin D, activation of pyroptosis inducer caspase-1 redirects cell fate toward caspase-3-dependent apoptosis in macrophages (79). Necroptosis effector protein receptor-interacting serine/threonine-protein kinase 3 (RIPK3) promotes cell death and NLRP3 inflammasome activation in the absence of mixed lineage kinase domain-like pseudokinase (MLKL) (80). These evidences collectively suggested that pyroptosis, necroptosis, and apoptosis cross-regulate each other. Here we found that treatments of Nlrp3 inhibitor OLT1177 not only suppressed ND-induced pyroptosis, but also apoptosis, necroptosis and autophagy levels are also suppressed (**Figure 3**). This is in agreement with our finding that treatments of Nlrp3 inhibitor OLT1177 suppressed of DENV-EIII-induced pyroptosis, necroptosis, and apoptosis in mouse platelets (38). Because the RCD pathways cross-regulate to each other, the CTS-RCDP could be served as a molecular-regulation fingerprint to identify the coordinated regulation of RCDs. For example, despite the detailed mechanism remains to be further studied, it seems that there are alternative RCDs, when one particular RCD is blocked. For instance, when the apoptosis (caspase-3) is blocked, pyroptosis levels are increased in the platelets (**Figures 7B, C**, ND + DEVD groups). When pyroptosis and apoptosis are blocked, ferroptosis levels are increased in the platelets (**Figures 3**, **5**, ferroptosis groups). These results suggested that RCDs are regulated in a coordinated manner. Here we identified pyroptosis and apoptosis as the top 2 ND-induced RCDs in platelets. ND-induced adverse effects, such as platelet aggregation and thrombocytopenia, may be therefore rescued through suppression of inflammasome activation and the cell death pathways. As a result, these inhibitors may be considered as antidotes for *in vivo* treated NDs. Notably, our analyses data revealed that P-selectin serves as an upstream pathway of Nlrp3 inflammasome and plays a critical role on the regulation of ND-induced platelet cell death.

P-selectin is a cell adhesion molecule expressing on the surfaces of activated endothelial cells and activated platelets. It is clear that P-selectin can function as a counter-receptor to stimulate P-selectin glycoprotein ligand-1 (PSGL-1) signaling, as interactions of PSGL-1 with immobilized P-selectin rapidly induce tyrosine phosphorylation of multiple proteins, and P-selectin-mediated adhesion enables activation outside-in signaling of β2 integrins in leukocytes (81). By contrast, the function of P-selectin in serving as a signaling receptor is less clear. We have previously shown that the binding of DENV-EIII or anti-P-selectin antibody to endothelial surface P-selectin initiate cellular inflammasome activation and pyroptosis (62). In agreement with this, here we found that blockage of ND-P-selectin interaction by addition of rP-sel markedly suppressed ND-induced platelet pyroptosis *in vitro* and *in vivo*. Moreover, compared to the wild type controls, P-selectin deficient mice displayed markedly less platelet activation and thrombocytopenia after ND injections. These results suggest that P-selectin is a ND-sensitizing pattern recognition receptor on platelets. Because P-selectin is highly expressed on the platelet surfaces during coagulation activation and various inflammatory diseases, the pattern-recognition property enables P-selectin serving as a critical coordinator that links the inflammation (immune system) to the thrombosis (coagulation system), and vice versa. The detailed mechanism of how P-selectin initiates inflammasome activation is worthy or further investigation. As the property of a blood-contacting material to induce thrombosis and inflammation determine the hemocompatibility, P-selectin-material interaction is one of the critical properties for analyzing hemocompatibility of *in vivo* used materials.

Previous reports have indicated that nanomaterial-induced NETs are critical for the initiation of adverse effects *in vivo* (74–76, 82). At the same time, platelets are involved in NET-related abnormal inflammation and coagulation (67, 83). Consequently, NETs may also contribute to ND-induced platelet-associated adverse effects *in vivo*. Our *in vitro* analysis results indicated that, compared to the supernatant from vehicle-treated control platelets, “PLT+ND sup” induced markedly higher NETosis levels of mouse neutrophils (**Figure 8**). Treatments of Nlrp3 inflammasome inhibitor OLT1177 drastically reduced NETosis-induction property of the “PLT+ND sup” (**Figure 8**), suggesting platelet pyroptosis and apoptosis are part of the up-stream pathways of ND-induced NETosis. At the same time, because treatments of NETosis inhibitor GSK484 markedly rescued ND-induced low platelet counts in mice (**Figure 8**), this indicated that NETosis in turn exacerbated ND-induced platelet defect. These results collectively suggested that there exists a feedforward regulation between platelets and neutrophils after ND-treatments. Moreover, in addition to direct activation of platelets, NDs can also indirectly cause platelet-associated defects through inducing NETosis. The interplay between platelets and neutrophils in ND-induced abnormal platelet responses are intriguing, and worth of further investigations.

In summary, here we found that treatments of 50 nm NDs with dose of 1.25 mg/kg can lead to platelet cell death and thrombocytopenia in mice. ND induced the platelet activation, pyroptosis and apoptosis through surface P-selectin-mediated activation of mitochondrial superoxide levels and Nlrp3 inflammasome. Blockage of P-selectin and Nlrp3 inflammasome by treatments of rP-sel and Nlrp3 inflammasome inhibitors markedly suppressed the adverse effects. However, NDs were shown to trigger the formation of platelet aggregates and NETs; and NDs are not easy to be sequestered *in vivo* and excreted from the body, because of their non-biodegradable property (74–76, 82). Consequently, despite of blockers of P-selectin and Nlrp3 inflammasome pathways displayed as antidotes of ND, these adverse effects prohibit the *in vivo* use of ND before the fundamental safety problems are solved.

## Data Availability Statement

The original contributions presented in the study are included in the article/**Supplementary Material**. Further inquiries can be directed to the corresponding author.

## Ethics Statement

The animal study was reviewed and approved by Prof. Kun-Ta Yang Department of Physiology, School of Medicine, Tzu Chi University, Hualien, Taiwan.

## Author Contributions

H-HC conceptualized and supervised this project. S-CH, L-CK, T-SL, H-SH, D-SS, and C-LC performed experiments and analysed the data. HHC wrote this manuscript. All authors contributed to the article and approved the submitted version.

## Funding

This work was supported by research funding from Ministry of Science and Technology, Taiwan (98-2320-B-320-004MY3, 101-2320-B- 320-004-MY3, 105-2923-B-320-001-MY3, 107- 2311-B-320-002-MY3), Tzu-Chi University (TCIRP95002; TCIRP98001; TCIRP101001) and Tzu-Chi Medical Foundation (TCMMP104; TCMMP108; TCMMP110; TCAS-108-01).

## Conflict of Interest

The authors declare that the research was conducted in the absence of any commercial or financial relationships that could be construed as a potential conflict of interest.

## Publisher’s Note

All claims expressed in this article are solely those of the authors and do not necessarily represent those of their affiliated organizations, or those of the publisher, the editors and the reviewers. Any product that may be evaluated in this article, or claim that may be made by its manufacturer, is not guaranteed or endorsed by the publisher.
