# Supplementary material for: Nanodiamond-Induced Thrombocytopenia in Mice Involve P-Selectin-Dependent Nlrp3 Inflammasome-Mediated Platelet Aggregation, Pyroptosis and Apoptosis
Source: Front Immunol. 2022 Apr 4;13:806686. doi: 10.3389/fimmu.2022.806686 (PMC9013758; doi:10.3389/fimmu.2022.806686)
Supplement: Supplementary file 1 [file DataSheet_1.docx]

Supplementary Material

**Nanodiamond-induced thrombocytopenia in mice involve P-selectin-dependent Nlrp3 inflammasome-mediated platelet aggregation, pyroptosis and apoptosis**

Shih-Che Hung^1^, Lu-Chu Ke^1^, Te-Sheng Lien^2^, Hsuan-Shun Huang^3^, Der-Shan Sun^1,2^, Chia-Liang Cheng^4^ and Hsin-Hou Chang^1,2*^

1. Institute of Medical Sciences, Tzu-Chi University, Hualien, Taiwan.
2. Department of Molecular Biology and Human Genetics, Tzu-Chi University, Hualien 970, Taiwan.
3. Center for Prevention and Therapy of Gynecological Cancers, Department of Research, Buddhist Tzu Chi General Hospital, Hualien 970, Taiwan
4. Department of Physics, National Dong Hwa University, Hualien 970, Taiwan.

*** Correspondence:**Hsin-Hou Chang
hhchang@gms.tcu.edu.tw; hhchang@mail.tcu.edu.tw

# Supplementary Figures S1-S8

**Figure S1**

**
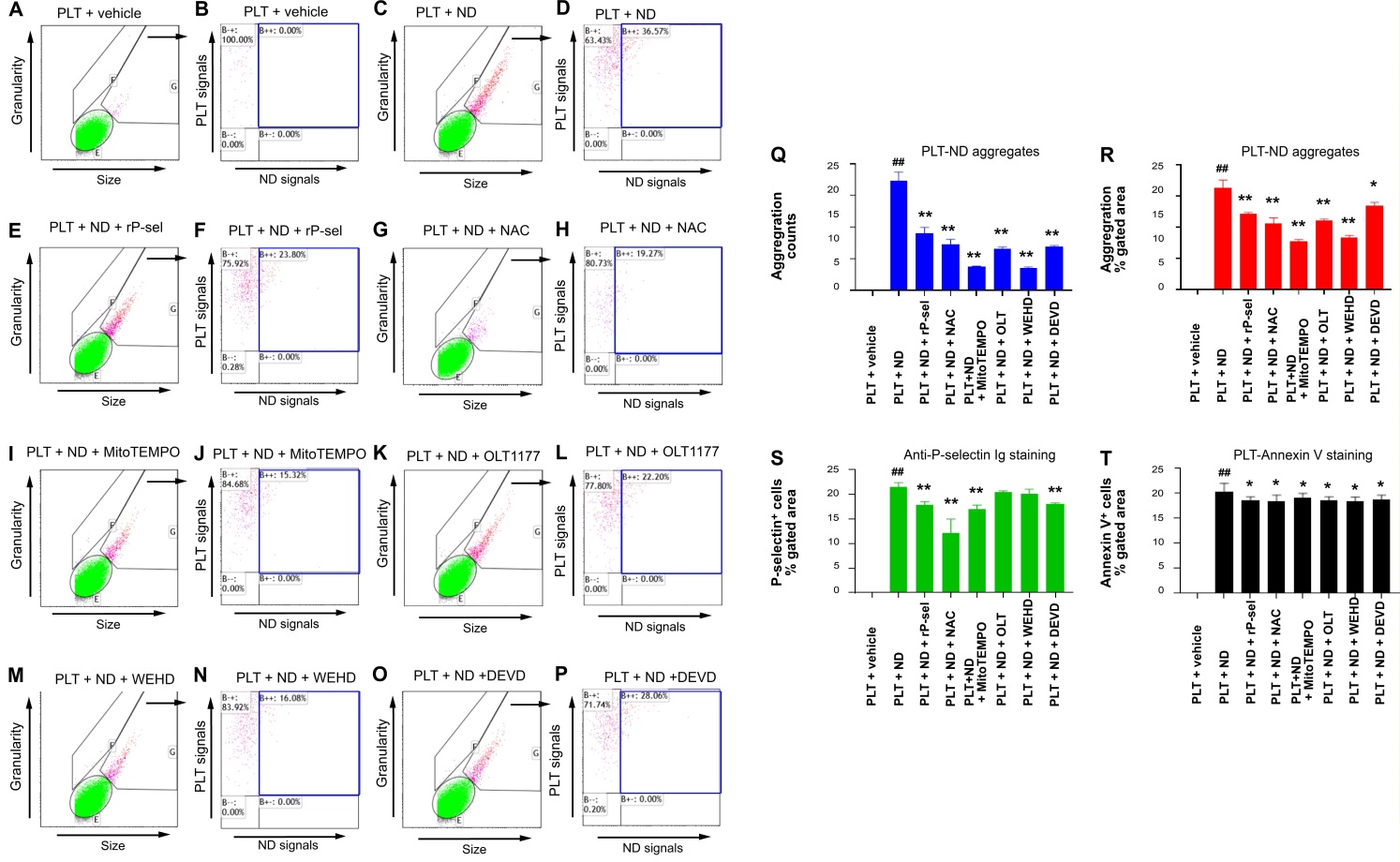
**

**Supplementary Figure 1. Flow cytometry gating strategy for analyzing nanodiamond (ND)-induced platelet aggregation.** CellTracker Blue Dye (ThermoFisher Scientific) was used to label mouse platelets, and then fluorescent labeled platelets (5 × 10^7^ /mL) were treated with red fluorescent 50 nm NDs (30 μg/mL, FND Biotech). Flow cytometry analyses were performed to investigate the aggregation levels of ND with platelets (PLTs) without (A, B: vehicle control; C, D: PLT + ND, no inhibitor control) or with inhibitors (E, F: rP-sel; G, H: NAC; I, J: MitoTEMPO; K, L: OLT; M, N: Z-WEHD-FMK; O, P: Z-DEVD-FMK) treatments. First we used size and granularity (A, C, E, G, I, K, M, O) to determine the larger-size aggregates. And then double confirmed levels of ND and platelet aggregations by measuring levels of ND and platelet double positive population (B, D, F, H, J, L, N, P: blue boxes; to exclude ND-ND or silica-silica beads aggregates). (Q-T) Quantitative results revealed that ND indeed induced platelet aggregation (Q, R), platelet surface P-selectin (S) and phosphatidylserine (T, annexin V binding) expressions (PLT + vehicle vs. PLT + ND groups). In addition, the inhibitor treatments markedly suppressed platelet ND-induced aggregation (Q, R), platelet surface P-selectin (S) and phosphatidylserine (T, annexin V binding) expressions in most of the analyzed groups, except OLT and Z-WEHD-FMK treatments on the suppression of platelet surface P-selectin expression (S, PLT + ND + OLT, and PLT + ND + WEHD groups). n = 3. (G) * *P* < 0.05, ** *P* < 0.01, vs. PLT + ND groups; ## *P* < 0.01 vs. PLT + vehicle groups.

**Figure S2**

**
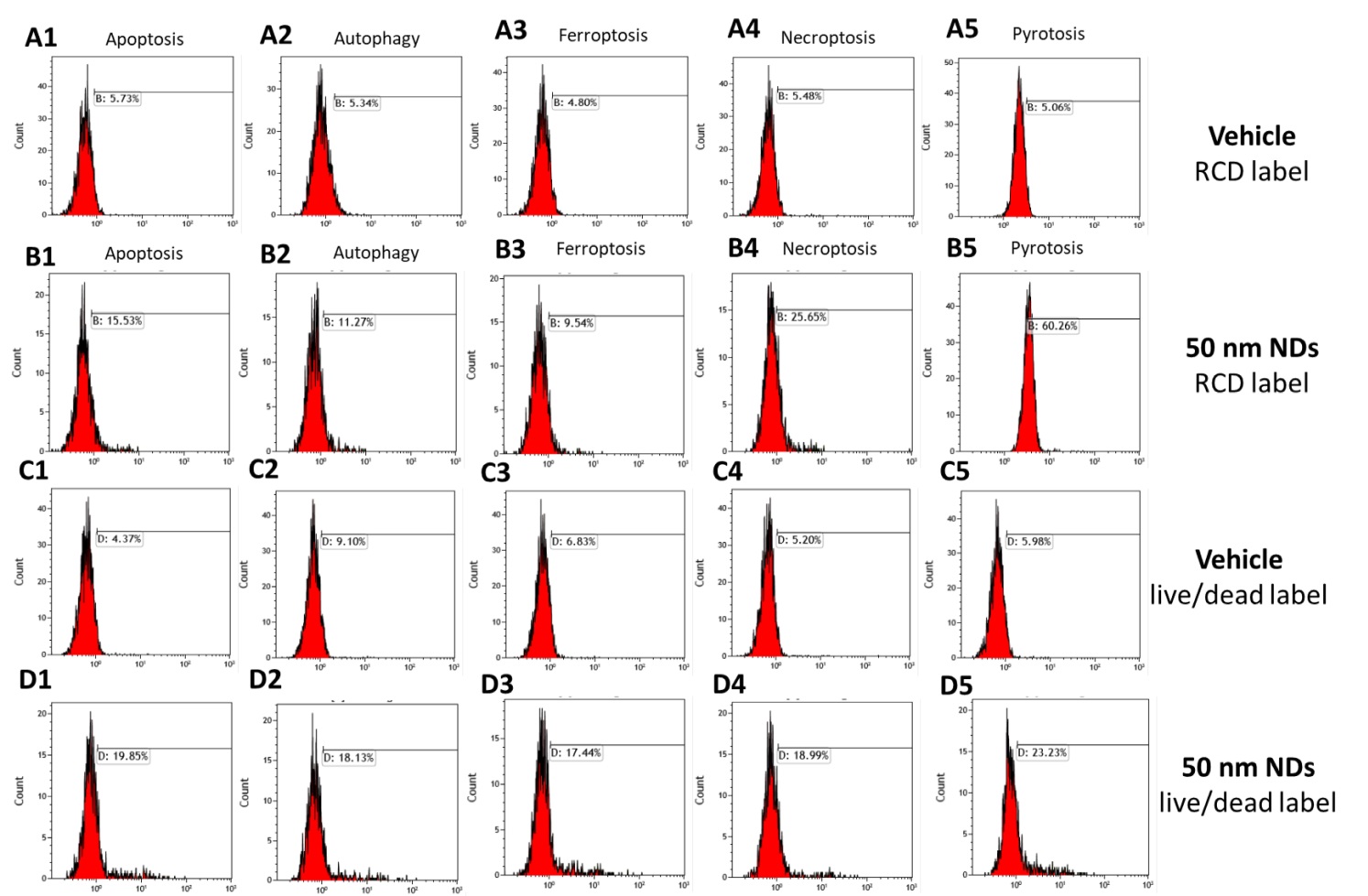
**

**Supplementary Figure 2. Calculation of respective percentage of nanodiamond (ND)-induced platelet regulated cell death (RCD).** According to previously reported methods (1-3), an example shows how respective RCD pathway percentages were determining by flow cytometry results. Because overlapping of detection wavelengths, it is not feasible to detect total 5 RCD pathways simultaneously in one cell-staining sample. Therefore, we performed double staining of respective RCD plus cell live and dead statue staining (an internal control), and then the RCD signals were only measured from the dead population. In brief, after platelet samples were treated with vehicle (A, C) or 50 nm NDs (B, D), these platelets were subjected to respective RCD (A, B) and cell-live/death status (C, D) staining. The respective increased cell death signal (e.g. apoptosis signal B1-A1; △apoptosis) was normalized (e.g. B1-A1/D1-C1; △ apoptosis/△total death cell) by increased death-cell population (e.g. D1-C1; △total death cell) of respective RCD staining. The sum of B1-A1/D1-C1, B2-A2/D2-C2, B3-A3/D3-C3, B4-A4/D4-C4 and B5-A5/D5-C5 was considered as 100%. As a result, the pyroptosis % was calculated as [B5-A5/D5-C5] / [(B1-A1/D1-C1) + (B2-A2/D2-C2) + (B3-A3/D3-C3) + (B4-A4/D4-C4) + (B5-A5/D5-C5)] × 100%, and is approximately 18%. Results showed in the figures 2D, 3 and 4, were obtained through this formula using averaged results from triplicated samples of each group.

**Figure S3**

**
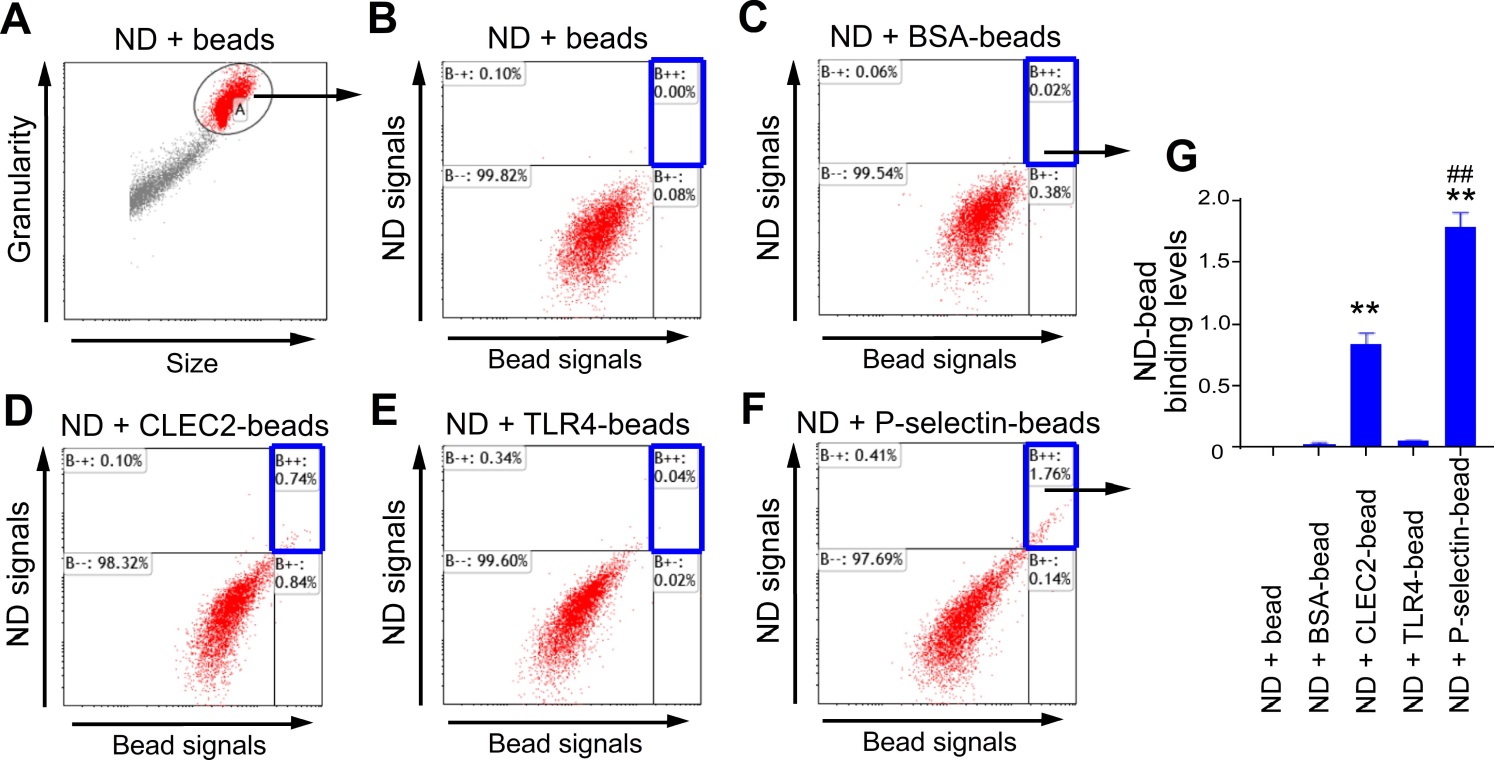
**

**Supplementary Figure 3. Binding properties of nanodiamond (ND) to various proteins.** (A-J) Using the gating strategy showed in A-F, flow cytometry analyses were performed to investigate the aggregation levels of ND with different proteins (A, B: ND + beads; C: ND + BSA beads; D: ND + CLEC2 beads; E: ND + TLR4 beads; F: ND + P-selectin beads) - coated fluorescent silica beads. First we used size and granularity (A) to determine the population with large-size aggregates. And then double confirmed levels of ND and protein-coated bead aggregations by measuring levels of ND and silica beads double positive population (B-F: blue boxes; to exclude ND-ND or silica-silica beads aggregates with increased sizes). Red fluorescent 50 nm NDs (FND Biotech) were used. (G) Quantitative results revealed that the binding property of P-selectin is markedly higher than the control protein BSA, while the other tested proteins such as CLEC2 and TLR4 are not. n = 6 (2 experiments with 3 samples per group). (G) ** *P* < 0.01, vs. BSA groups; ## *P* < 0.01 vs. ND + CLEC2 beads groups.

**Figure S4**

**
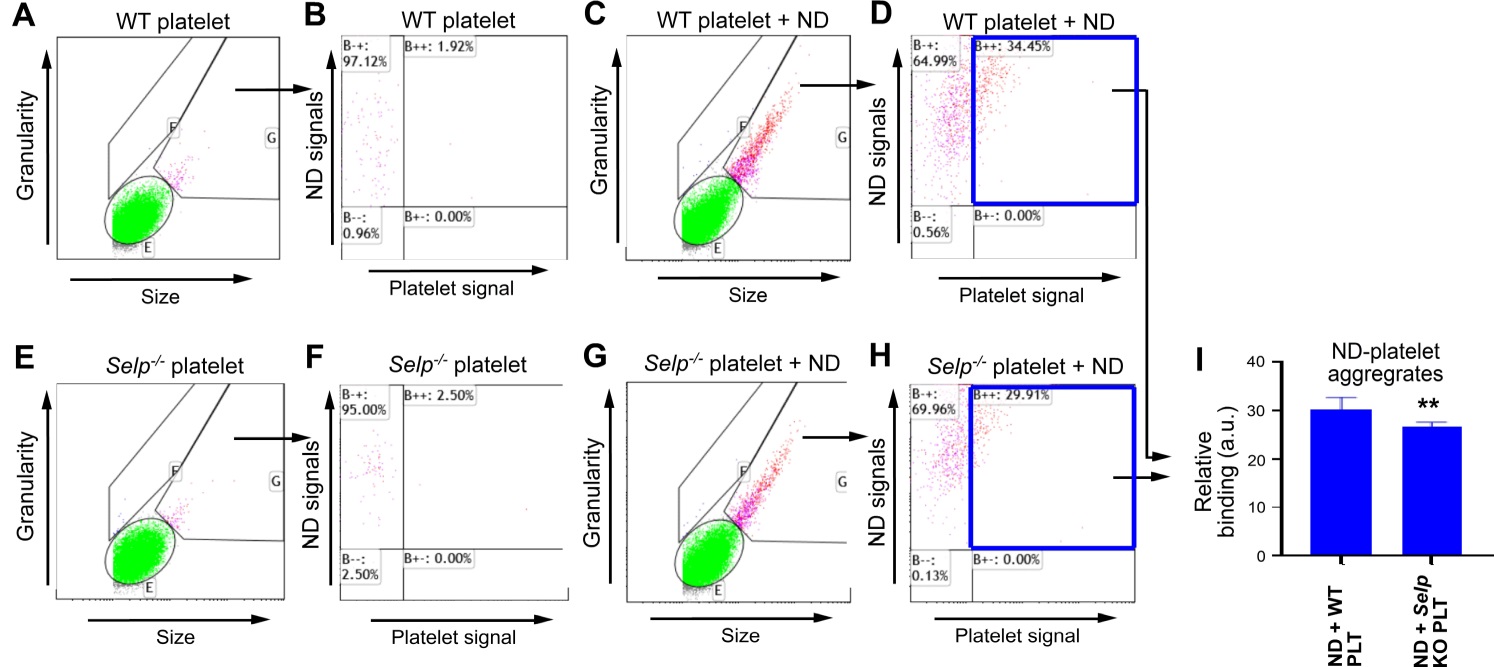
**

**Supplementary Figure 4. Binding properties of nanodiamond (ND) to wild type and P-selectin deficient platelets.** CellTracker Blue Dye (ThermoFisher Scientific) was used to label mouse platelets, and then fluorescent labeled platelets (5 × 10^7^ /mL) were treated with red fluorescent 50 nm NDs (30 μg/mL, FND Biotech). (A-H) Using the gating strategy showed in A-H, (A-D: wild type platelets; E-H: *Selp^-/-^* mutant platelets; A, B, E, F: no ND vehicle controls; C, D, G, H: platelets + ND groups), flow cytometry analysis revealed that the aggregation levels of ND to platelet with or without P-selectin expression (D, H: blue boxes, double positive population; to exclude ND-ND or platelet-platelet aggregates with increased sizes). (I) In agreement with the P-selectin protein binding experiments, the binding levels of NDs to wild type (*Selp^+/+^*) platelets are markedly higher than the binding levels to platelets from P-selectin deficient *Selp^-/-^* mutant mice. n = 3. (I) * *P* < 0.05, vs. BSA groups; (F) ** *P* < 0.01 vs. ND + wild type (WT) platelet (PLT) groups. Carboxyfluorescein succinimidyl ester (CFSE) was used to label the mouse platelets.

**Figure S5.**

**
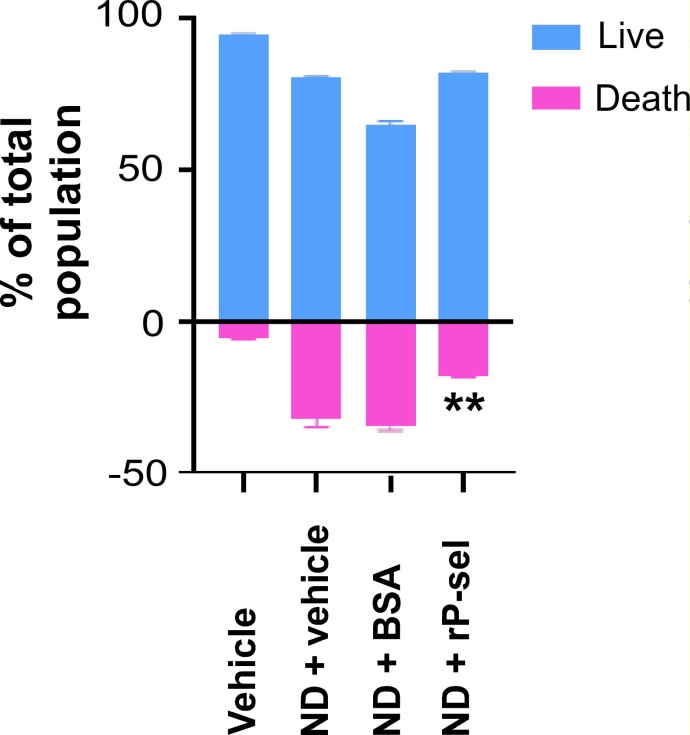
**

**Supplementary Figure 5.** **Addition of recombinant soluble P-selectin (rP-sel) but not bovine serum albumin (BSA) rescues nanodiamond (ND)-induced platelet cell death.** Washed mouse platelets were treated with NDs without or with additional protein BSA or rP-sel treatments *in vitro*. Treatments of rP-sel but not BSA showed rescue effect on ND-induced cell death. n = 6 (3 experiments with 2 samples per group). ## *P* < 0.01 significantly increased vs. ND + vehicle groups; * *P* < 0.05 significantly suppressed vs. ND + vehicle groups.

**Figure S6.**

**
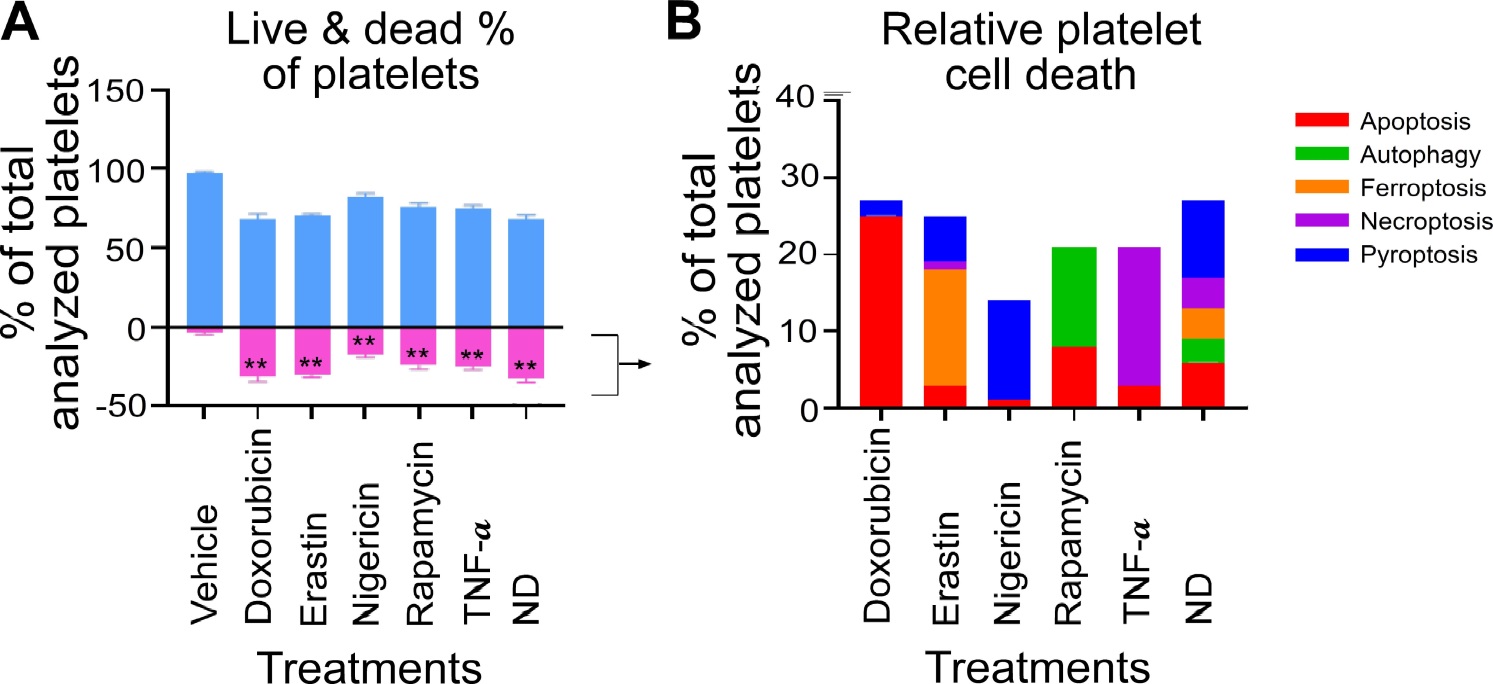
**

**Supplementary Figure 6.** **ND and specific cell death inducers-induced RCD in platelets.** (A) Washed mouse platelets treated with vehicle, and various specific cell death inducers and NDs (30 ng/mL); the live and dead cell populations were estimated using Zombie-NIR Kit labeling and flow cytometry analysis. (B) RCD inducers, doxorubicin (DOX; apoptosis) (2.5 μg/mL), rapamycin (autophagy) (0.5 μM), erastin (ferroptosis) (10 μM), TNF-α (necroptosis) (2.5 ng/mL), and nigericin (pyroptosis) (3.5 μM) induced relatively simple RCD patterns. By contrast, ND induced multiple RCD pathways, in which pyroptosis and apoptosis are the top 2 major RCD responses (B). ***P* < 0.01 vs. vehicle groups. n = 6 (3 experiments with 2 samples per group).

.

**Figure S7.**

**
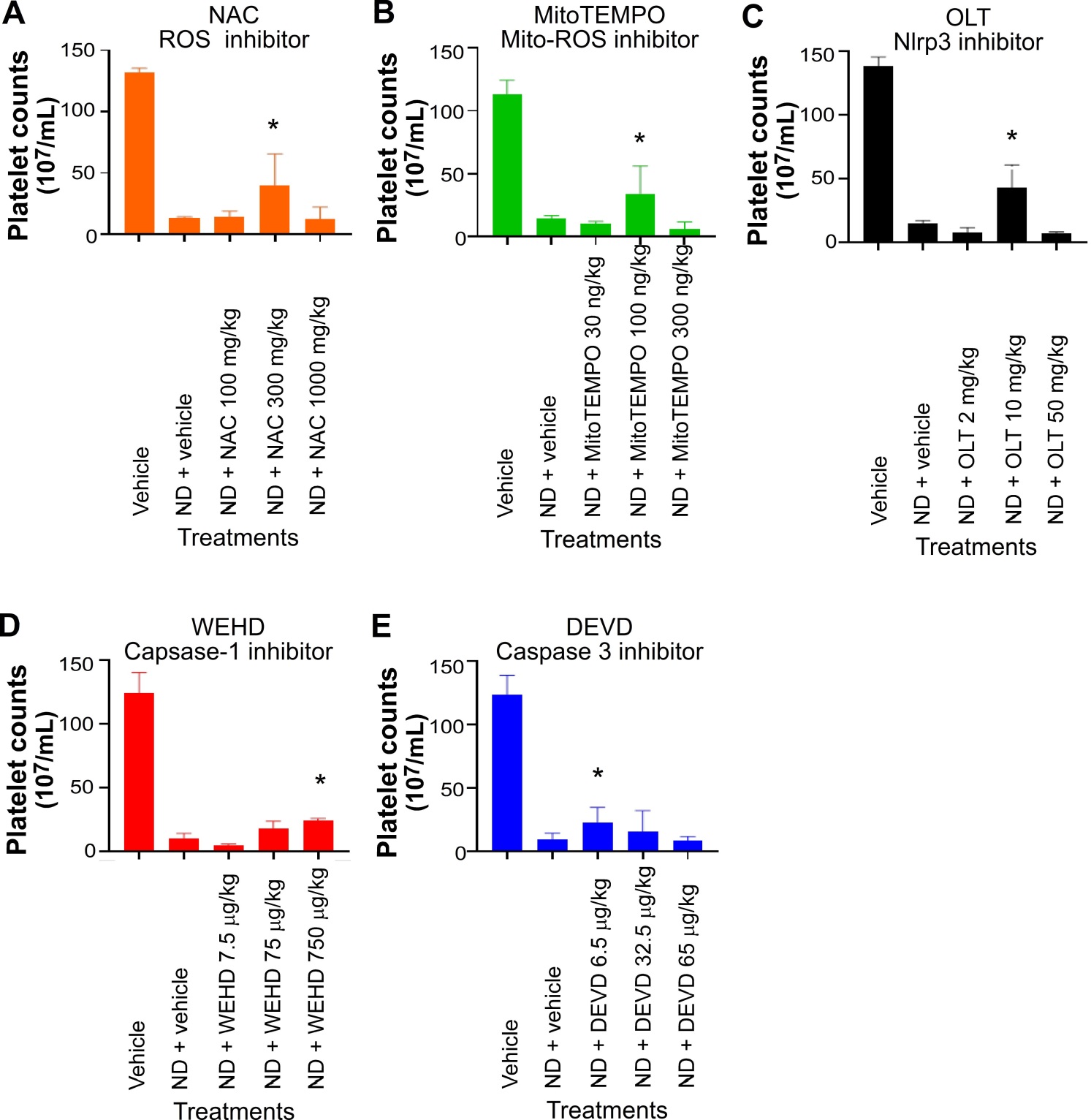
**

**Supplementary Figure 7.** **Rescue of ND-induced thrombocytopenia by treatments of inhibitors against P-selectin, ROS, Nlrp3 inflammasome and caspase-3 pathways in mice.** Platelet counts were analyzed after wild type mice subjected to various treatments for 24 h. We found that ND-induced thrombocytopenia could be rescued by treatments of NAC (A), MitoTEMPO (B), OLT1177 (C, OLT), Z-WEHD-FMK (D) and Z-DEVD-FMK (E). n = 6 (2 experiments with 3 mice per group); * P < 0.05, vs. ND + vehicle groups.

**Figure S8.**

**
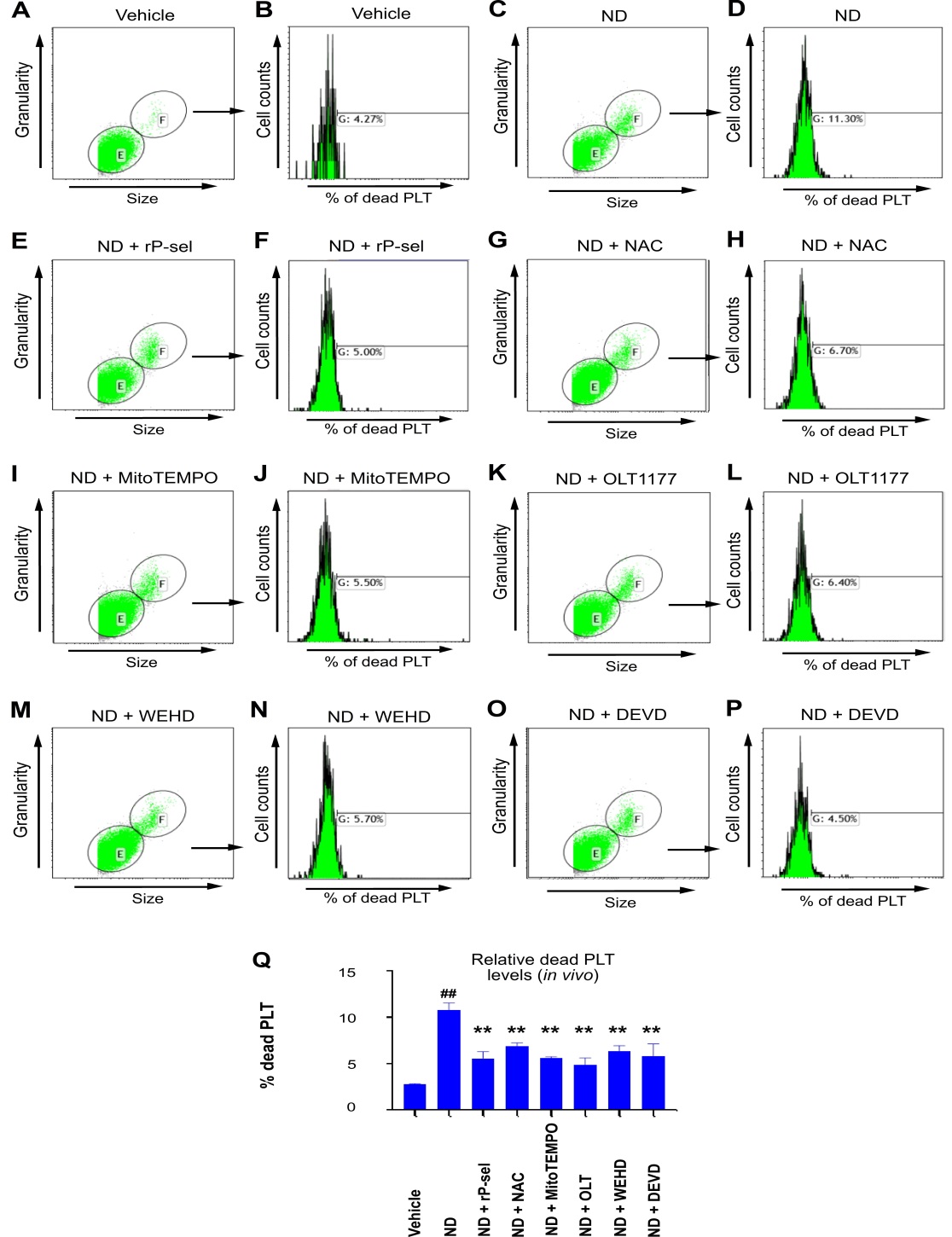
**

**Supplementary Figure 8.** **Rescue of ND-induced aggregation-associated platelet cell death by treatments of inhibitors against P-selectin, ROS, Nlrp3 inflammasome and caspase-3 pathways in mice.** Platelets were isolated and analyzed after wild type mice subjected to various treatments for 24 h. First we used size and granularity and size (A, C, E, G, I, K, M, O) to determine the larger-size aggregates; example gating conditions are showed (A-P). And then the platelet cell death levels were estimated using Zombie-NIR Kit labeling. (Q) Quantitative results revealed that the platelet cell death levels in the ND treated groups (C, D, Q, ND groups) are markedly higher than the vehicle treated control groups (A, B, Q vehicle groups). In addition, inhibitors including rP-sel (E, F), NAC (G, H), MitoTEMPO (I, J), OLT (K, L), Z-WEHD-FMK (M, N) and Z-DEVD-FMK (O, P) were all effectively suppressed ND-induced aggregation-associated platelet cell death (Q). (G) ** *P* < 0.01, vs. ND groups; ## *P* < 0.01 vs. vehicle groups. n = 6 (2 experiments with 3 mice per group).

**References**

1. Lien TS, Sun DS, Wu CY, Chang HH. Exposure to Dengue Envelope Protein Domain III Induces Nlrp3 Inflammasome-Dependent Endothelial Dysfunction and Hemorrhage in Mice. *Front Immunol* (2021) 12:617251.

2. Lien TS, Sun DS, Hung SC, Wu WS, Chang HH. Dengue virus envelope protein domain III induces Nlrp3 inflammasome-dependent NETosis-mediated inflammation in mice. *Front Immunol* (2021) 12:618577.

3. Lien TS, Chan H, Sun DS, Wu JC, Lin YY, Lin GL, et al. Exposure of Platelets to Dengue Virus and Envelope Protein Domain III Induces Nlrp3 Inflammasome-Dependent Platelet Cell Death and Thrombocytopenia in Mice. *Front Immunol* (2021) 12:616394.
